# Expression Profiling Using a cDNA Array and Immunohistochemistry for the Extracellular Matrix Genes FN-1, ITGA-3, ITGB-5, MMP-2, and MMP-9 in Colorectal Carcinoma Progression and Dissemination

**DOI:** 10.1155/2014/102541

**Published:** 2014-03-04

**Authors:** Suzana Angelica Silva Lustosa, Luciano de Souza Viana, Renato José Affonso, Sandra Regina Morini Silva, Marcos Vinicius Araujo Denadai, Silvia Regina Caminada de Toledo, Indhira Dias Oliveira, Delcio Matos

**Affiliations:** ^1^Postgraduate Program in Interdisciplinary Surgery Science, UNIFESP-Escola Paulista de Medicina, São Paulo, SP 04023-062, Brazil; ^2^Research and Teaching Unit of Hospital Municipal Dr. Munir Rafful, Volta Redonda, RJ 27277-130, Brazil; ^3^Hospital de Cancer de Barretos-Fundação Pio XII, Barretos, SP 14784-400, Brazil; ^4^Genetics Laboratory-GRAAAC, UNIFESP-Escola Paulista de Medicina, São Paulo, SP 04023-062, Brazil

## Abstract

Colorectal cancer dissemination depends on extracellular matrix genes related to remodeling and degradation of the matrix structure. This investigation intended to evaluate the association between FN-1, ITGA-3, ITGB-5, MMP-2, and MMP-9 gene and protein expression levels in tumor tissue with clinical and histopathological neoplastic parameters of cancer dissemination. The expression associations between ECM molecules and selected epithelial markers EGFR, VEGF, Bcl2, P53, and KI-67 have also been examined in 114 patients with colorectal cancer who underwent primary tumor resection. Quantitative real-time PCR and immunohistochemistry tissue microarray methods were performed in samples from the primary tumors. The gene expression results showed that the ITGA-3 and ITGB-5 genes were overexpressed in tumors with lymph node and distant metastasis (III/IV-stage tumors compared with I/II tumors). The MMP-2 gene showed significant overexpression in mucinous type tumors, and MMP-9 was overexpressed in villous adenocarcinoma histologic type tumors. The ECM genes MMP9 and ITGA-3 have shown a significant expression correlation with EGFR epithelial marker. The overexpression of the matrix extracellular genes ITGA-3 and ITGB-5 is associated with advanced stage tumors, and the genes MMP-2 and MMP-9 are overexpressed in mucinous and villous adenocarcinoma type tumors, respectively. The epithelial marker EGFR overactivity has been shown to be associated with the ECM genes MMP-9 and ITGA-3 expression.

## 1. Introduction

Studies have shown that alterations in genes that regulate basic cell functions such as cell-cell adhesion and ECM-cell adhesion are followed by penetration of the basal membrane, destroying the physical structure of the tissue [1]. Alterations in the expression of adhesion molecules can influence tumor aggression resulting in local infiltrative growth and metastasis. Thus, the basal membrane and the ECM jointly represent two important physical barriers to malignant invasion, and their degradation by metalloproteinase enzymes may have an important role in tumor progression and metastatic dissemination [2–4]. Other researchers, however, have reported that, in general, the expression levels of integrins alpha 3 and alpha 5 are reduced in many colorectal carcinomas (CRCs) [5, 6].

Some authors have recently demonstrated that integrin inhibition, at any point of action, may lead to tumor progression inhibition. Therefore, integrin inhibition may represent a pharmacological target for cancer treatment and prevention through the suppression of cell migration and invasion and, following apoptosis induction, also through blocking tumor angiogenesis and metastases [7].

In most human cancers, the metalloproteinase expression and activity levels are high compared with normal tissue, and this has also been demonstrated in colorectal adenocarcinomas [8, 9]. From these results, several researchers have analyzed the possibility that metalloproteinase expression and activity levels can be used as tumor markers, aiming to prevent tumor growth, invasion, and metastasis [10, 11].

Studies have explored the hypothesis that the MMP-9 functions as a key regulator of the malignant phenotype in patients with colorectal tumors presenting with overexpression of this protease relative to the adjacent normal tissues. In this context, MMP-9 is the main agent of cancer cell invasion and metastasis in the epithelial and stromal cells of the primary colorectal tumor. In addition, human colorectal cancer cells have the ability to synthesize and secrete MMP-9. This effect, associated with the induction of proteolytic functions in the pericellular space, causes metastasis development. Hence, the MMP-9 present in tumor epithelial cells can represent a specific target for the diagnosis and treatment of metastatic CRC.

Recently, Viana et al. reported that the expression of the genes SPARC, SPP1, FN-1, ITGA-5, and ITGAV correlates with common parameters of progression and dissemination in CRC, and overexpression of the ITGAV gene and protein correlates with an increased risk of perineural invasion. Moreover, according to these authors, the strong correlation of IHC expression between ITGAV and EGFR suggests an interaction between these two signaling pathways [12]. Denadai et al., in 2013, also showed that increased expression levels of ITGA-6 and ITGAV are related to venous invasion and neural infiltration, respectively, while overexpression of ITGB5 and ITGA3 is associated with stage III (TNM), and overexpression of ITGA-5 correlates with the presence of mucinous-type malignant neoplasias [13]. The authors concluded that follow-up studies, preferably with a controlled prospective design, are necessary to establish the roles of such genes as potential biomarkers to predict disease extent or outcome and possibly contribute to the management of CRC patients.

According to Nowell, in 2002, tumors become more clinically and biologically aggressive over time and this has been termed “tumor progression” and includes, among other properties, invasion and metastasis, as well as more efficient escape from host immune regulation. Molecular techniques have shown that tumors expand as a clone, from a single altered cell and sequential somatic genetic changes, generating increasingly aggressive subpopulations within the expanding clone. So far multiple types of genes have been identified, and they differ in different tumors, but they provide potential specific targets for important new therapies [14].

This study aimed to evaluate the relationship of the expression levels of select ECM genes and proteins, FN-1, ITGA-3, ITGB-5, MMP-2, and MMP-9, with CRC progression and dissemination and with that of P53, Bcl-2, KI-67, EGFR, and VEGF, as it has been shown by several authors that proliferation, apoptosis, and cell migration are regulated by cell-cell interaction and extracellular matrix cell components. It is also worth noting that the growth factors EGF and VEGF are usually stored in the ECM and can be activated and released after ECM modulation [15–17].

## 2. Methods

### 2.1. Patients and Tumor Samples

We studied 114 patients with stage I–IV CRC who underwent primary tumor resection at the Fundação Pio XII, Barretos Cancer Hospital, between August 2006 and July 2009. All patients were eligible for the analysis of the expression of the genes of interest through real-time PCR and immunohistochemistry (IHC) assays using the tissue microarray (TMA) technique. The median followup was 30 months at the time of this report.

The ethical use of human tissue for research was approved by the institutional review board, and the design of this study followed the principles of the Declaration of Helsinki and also complied with the principles of good clinical practice. This study was also approved by the Ethics Committee of the Barretos Cancer Hospital and UNIFESP-Escola Paulista de Medicina, São Paulo, Brazil.

In this study, we included patients of both genders whose age was >18 years. The patients who had received neoadjuvant treatment (chemotherapy or radiotherapy) were excluded. In all patients, tumor tissue was sampled during surgery and cryopreserved, and paraffin blocks were available for further histopathological analysis. The patients without primary CRC site resection were excluded as well as patients with a previous or current diagnosis of another primary malignancy in any location of the body other than nonmelanoma skin cancer or in situ carcinoma of the cervix. The patients with a known history of familial CRC were also excluded. The chromosomal and microsatellite instability statuses were not assessed.

Sixty-three patients were male (55.3%), and 51 were female (44.7%). The median patient age was 60 years (24–83); 58 patients (50.9%) were over 60 years of age. Concerning the location of the primary tumor, the right colon was affected in 41 cases (36.0%) and the left colon in 41 cases (36.0%), and the rectum was the primary tumor site in 32 cases (28.0%). Twenty-five (21.9%) patients were considered as TNM stage I, 39 (34.2%) as TNM stage II, 34 (29.8%) as TNM stage III, and 16 (14.0%) as TNM stage IV. The most frequent site for metastasis was the liver (9 patients) followed by the peritoneum (3 patients), lungs (2 patients), and ovary (2 patients).  Table 1 shows the distribution of patients according to the covariable categorization.

### 2.2. Outcome Measures

The patients were classified according to the following clinical and pathological characteristics: age group (<60 or >60 years), gender (male versus female), site of the primary tumor (right colon versus left colon versus rectum), histological classification (adenocarcinoma not otherwise specified versus mucinous adenocarcinoma), tumor grade (low (grades I and II) versus high (grades III and IV)), and peritumoral lymphocyte infiltration (presence versus absence).

Histological characteristics commonly associated with tumor dissemination and progression have been categorized as follows: venous invasion (presence versus absence); lymphatic vessel invasion (presence versus absence); perineural invasion (presence versus absence); degree of tumor invasion into the organ wall (T 1-2 versus T 3-4, AJCC 2002, 6th edition); lymph node metastasis (presence versus absence); distant metastases (presence versus absence); and TNM staging (I-II versus III-IV, AJCC 2002, 6th edition).

We hypothesized that ECM molecules may be associated with CRC progression and dissemination; therefore, differences in ECM marker expression with respect to the categorization of one of the histological covariates mentioned above were analyzed using both reverse transcription- (RT-) PCR and TMA.

### 2.3. RNA Extraction and cDNA Synthesis by RT-PCR

Cryopreserved samples were embedded in medium for frozen tissue specimens (Tissue-Tek OCT; Sakura Finetek, Torrance, CA, USA) and fitted into a cryostat (CM1850 UV; Leica Microsystems, Nussloch, Germany) for histological analysis. The slides mounted with sections of 4 *μ*m thickness were subjected to hematoxylin-eosin staining (Merck, Darmstadt, Germany) and analyzed by a pathologist to ensure that the selected samples represented the general tumor histology and were free of necrosis or calcifications.

The areas of interest were identified microscopically and marked for macrodissection. These slides were used as guides to select and cut tissues in the cryostat. For each sample, sterile individual scalpel blades were used. After discarding areas inappropriate for RNA extraction, the tissue was mechanically macerated with liquid nitrogen and transferred to 1.5 mL RNase- and DNase-free microtubes containing 1,000 *μ*L TRIzol (Invitrogen, Carlsbad, CA, USA). RNA was extracted according to the manufacturer's instructions, and RNA quantification was performed using a spectrophotometer (Thermo Scientific NanoDrop 2000). The quality and integrity of the RNA were verified by the presence of 28S and 18S bands in an agarose gel and stained with 1% ethidium bromide. RNA was purified using the RNeasy mini kit (Qiagen, Valencia, CA, USA) following the manufacturer's recommendations, diluted with 30 mL of water free of RNase and DNase (Qiagen), quantified spectrophotometrically at a wavelength of 260 nm (NanoVue; GE Healthcare, Chicago, IL, USA), and stored at –80°C until use. RT-PCR was performed using the Super-Script TM III first-strand synthesis SuperMix (Invitrogen), as recommended by the manufacturer. The reaction was performed in a 20 *μ*L final volume containing 2 *μ*g of total RNA with oligo (dT)_20_ as a primer. The transcription phase was performed in a thermal cycler (Mastercycler_ep Gradient S; Eppendorf, Hamburg, Germany), and the cDNA was stored at –20°C for future reactions.

### 2.4. Analysis of the Genes of Interest

For each sample, an ECM and adhesion molecule PCR array (PAHS-013; SABiosciences, Qiagen) plate was used. A mixture was prepared containing 1,275 *μ*L of buffer with SYBR Green (2x Master Mix SABiosciences RT2 qPCR), 1,173 *μ*L RNase-free H_2_O, and 102 *μ*L of the cDNA sample. Next, 25 *μ*L aliquots were added to each well of the 96-well plate. The reactions were performed in a thermal cycler (ABI 7500; Applied Biosystems, Foster City, CA, USA), according to the following protocol: 95°C for 10 min and 40 cycles at 95°C for 15 s and 60°C for 1 min. Data analysis was performed using method from the website http://pcrdataanalysis.sabiosciences.com/pcr/arrayanalysis.php.

Gene expression was classified as “high” or “low,” considering the level of expression obtained after grouping patients by the covariates of interest; that is, after categorizing patients into the control or interest groups according to the covariates studied, gene expression was determined in both groups.

### 2.5. TMA Block Construction

Original paraffin blocks were sectioned at a 4 *μ*m thickness and stained with hematoxylin-eosin. All sections were reviewed to confirm the CRC diagnosis, and the histopathologic findings were reevaluated.

A map was prepared using a spreadsheet containing the locations and identification of tissue samples for the construction of the TMA block. The map also guided further readings of the IHC reactions. With the aid of Beecher TM equipment (Beecher Instruments, Silver Spring, MD, USA), the TMA blocks were prepared according to the manufacturer's specifications in the following steps: marking of the selected area in the respective paraffin block; use of the equipment to create a hollow space in the recipient block; extraction of a cylindrical tissue of the donor block, measuring 1 mm in diameter of the previously selected respective area of interest; transfer of the cylindrical tissue obtained from the donor block to the hollow space previously created in the recipient block; progression, in fractions of millimeters, to new positions within the recipient block, thereby creating a collection of tissue samples following a matrix arrangement; and assessment of the final quality of the block for storage.

For adhesion of the TMA block section onto the slides, an adhesive tape system was used (Instrumedics, Hackensack, NJ, USA). The samples were cut to a thickness of 4 *μ*m, and a small roll was used to press the section on the tape. The tape with the attached histological section was then placed on a resin-coated slide (part of the adhesive system kit) and pressed with the same roll for better adherence. Afterwards, the slides were placed under UV light for 20 min and were posteriorly exposed to a solvent solution (TPC) for 20 additional minutes. The slides were dried, and the tapes were removed. Afterwards, the slides were paraffin-embedded and sent for storage in ideal cooling conditions.

### 2.6. IHC Technique

The TMA block sections were mounted onto glass slides coated with silane (3-aminopropyltriethoxysilane) and dried for 30 min at 37°C. The paraffin was removed with xylene and rehydrated through a series of graded alcohols. Endogenous peroxidase activity was blocked by incubating the sections in a methanol bath containing 3% hydrogen peroxide for 20 min, followed by washing in distilled water. The sections were initially submitted to heat-induced epitope retrieval using citrate buffer (pH 9.0) in an uncovered pressure cooker (Eterna; Nigro, Araraquara, Brazil). The slides were immersed in the buffer solution, and the pressure cooker was closed with the safety valve open. Once the saturated steam was released, the safety valve was lowered until full pressurization was achieved. After 4 min under full pressurization, the closed pressure cooker was placed under running water for cooling. After removing the lid, the slides were washed in distilled running water.

Blocking of the endogenous peroxidase was obtained with 3% H_2_O_2_ (10 vol), with 3 washes of 10 min duration each. The slides were again washed in distilled running water and then in phosphate-buffered saline (10 mM; pH 7.4) for 5 min. Afterwards, the primary antibody was applied, and the slides were incubated overnight at 8°C.

### 2.7. Primary Antibodies

The primary monoclonal antibodies used were obtained from ABCAM Inc. (Cambridge, MA, USA) and were as follows: anti-integrin alpha 3 that reacts with human (IHC-FoFr or Fr) and mouse IgG1 isotypes (clone F35 177-1; 100 *μ*g; ab 20140); anti-integrin beta 5 that reacts with human (IHC-P or Fr) and mouse IgG2a isotypes (ab 93943); anti-MMP-2, rabbit IgG isotype (ab52756); and anti-MMP-9 (ab76003; clone EP1254). All antibodies were used at a 1 : 400 dilution.

In addition, the following non-ECM primary antibodies were used in this study: anti-p53 (IgG2b class; clone DO-7; 1 : 300; M7001; DAKOCytomation, Glostrup, Denmark); anti-Bcl-2 (mouse isotype IgG1; clone 124; 1 : 600; M0887; DAKOCytomation); anti-VEGF (mouse IgG1 isotype; clone VG 1; 1 : 100; M7273; DAKOCytomation); anti-KI-67 (mouse isotype IgG1; clone MIB-1; 1 : 500; M7240; DAKOCytomation); and anti-EGFR (mouse IgG1 isotype; clone EGFR-25; NCLEGFR-384; Novocastra, Newcastle, UK). The positive controls used for IHC analysis were normal human kidney tissue for FN-1, human tonsils for ITGA-3, ITGB-5, VEGF, KI-67, P53, and Bcl-2, and placenta for EGFR.

### 2.8. Immunostaining Analysis

A preliminary test was performed to identify the optimal antibody concentration and to select positive and negative controls using the dilution data supplied by the manufacturer. After washing the primary antibody with phosphate-buffered saline, the slides were incubated with biotin-free polymer in the Advance TM visualization system (DAKO) for 30 min. A freshly prepared solution containing 1 drop of 3,3′-diaminobenzidine tetrahydrochloride (DAB; Sigma, St. Louis, MO, USA) with 1 mL of substrate (DAKO) was applied for 5 min on each slide. The DAB solution was removed by washing with distilled water. The slides were counterstained with hematoxylin, dehydrated in ethanol, cleared in xylene, and mounted using Entelan TM [18–20].

Tissue expression of FN-1, ITGA-3, ITGB-5, MMP-1, and MMP-9 was categorized dichotomously as “high expression” or “low expression,” according to the “quick score” method [21, 22]. This score system uses a combination of the percentage of stained cells (*P*) and staining intensity (*I*), and the “quick score” was calculated by multiplying both values.

The scores used for the percentage of stained tumor cells were as follows: 0 points (absence of stained cells); 1 point (>25% of stained cells); 2 points (26–50% of stained cells); and 3 points (>50% of stained cells). The scores used for the staining intensity were as follows: 1 point (mild intensity); 2 points (moderate intensity); and 3 points (intense staining). As a result, expression of a gene product in tumor cells was considered to be high (overexpressed) when the final score was >4 (*P* × 1 >4), and the markers that presented a final score <4 were considered to have low expression. The stroma and the tumor cells were not treated separately during IHC analysis, and only the level of expression of markers on tumor cells was considered for scoring.

The validation of different expression levels of the genes detected by real-time PCR analysis was performed by the verification of the protein expression related to each gene by IHC. Thus, for each gene (fibronectin, integrins, and metalloproteases) with increased or reduced expression by array tracing, the corresponding protein was analyzed by the antigen-antibody reaction (IHC) in TMA slides. The confirmation of the protein expression increase by IH validates the molecular finding of the tracing by RT-PCR.

### 2.9. Statistical Analyses

Statistical associations between gene and protein expression levels of FN-1, ITGA-3, ITGB-5, MMP-2, and MMP-9 and the clinicopathological factors were determined using a nonparametric Mann-Whitney *U* test for quantitative variables and a chi-square test for qualitative variables, that is, frequencies and proportions. When the chi-square test assumptions were not met, Fisher's exact test was used. To measure the association between the ECM markers FN-1, ITGA-3, ITGB-5, MMP-2, and MMP-9 and the non-ECM markers EGFR, VEGF, P53, Bcl-2, and KI-67 (ordinal variables), the Spearman correlation coefficient was used [23].

The significance level was set at 5% (*P* < 0.05), and the data were analyzed using Statistical Package for Social Sciences (SPSS) software (Chicago, IL, USA), version 15.0. The Shapiro-Wilk test was used to verify that the data had a normal distribution.

## 3. Results

### 3.1. FN-1, ITGA-3, ITGB-5, MMP-2, and MMP-9 ECM Gene Expression Levels

The expression levels of the genes of interest according to the covariates studied through real-time PCR showed low expression of the FN-1 gene in patients <60 years of age compared with those ≥60 years of age (*P* = 0.022).

The ITGA-3 and ITGB-5 gene expression levels in the tumor tissue as determined using RT-PCR were not considered significant when analyzed with regard to the different measures of tumor dissemination outcome, except for those related to TNM staging and cell differentiation degree.

ITGA-3 gene expression showed a significance level of *P* = 0.016 and a *fold regulation* of 2.58 comparing TNM III, IV versus TNM I, II stages. With regard to the ITGB-5 gene, a reduction in the group expression of III cell differentiation degree was observed when compared with the GI and GII group (*P* = 0.04 and a *fold change* of –2.11) and an increase of this gene expression in the tumors of TNM III, IV versus TNM I, II stages (*P* = 0.029 and a *fold change* of 1.33).  Table 2 shows the distribution of the significant results of the RTPCR expression of the genes of interest.

The expression levels of MMP2 and MMP9 genes in the tumor tissue as determined using RT-PCR were considered significant when analyzed with regard to the different measures of tumor dissemination outcome, except for those related to mucinous and villous histological types and in the parameter of venous invasion dissemination. Therefore, MMP2 gene expression was significantly different between mucinous and nonmucinous carcinomas (*P* = 0.001) and in patients aged over 60 years (*P* < 0.0001). With regard to the tumor expression of the MMP9 gene, an increase of this gene expression was noted in tumors with TNM III and IV staging compared with TNM I and II staging (*P* = 0.0001), in venous invasion tumors compared with those without venous invasion (*P* < 0.001), and in carcinoma tumors with a villous component compared with carcinomas without a villous component (*P* < 0.0001). Tables 3, 4, 5, 6, 7, 8, and 9 show the results of immunohistochemical expression of the EMC genes and the non-ECM molecular markers according to the outcome measures degree of tumor cell differentiation, tumor TNM classification, peritumoral lymphocytic infiltration, venous invasion, perineural invasion, and type of tumor (tubular, mucinous, and villous).

Table 10 shows the distribution in absolute numbers and percentages by IHC of proteins corresponding to the ITGB-5, ITGA-3, MMP-2, MMP-9, and FN-1 ECM genes and the EGFR, VEGF, KI-67, P53, and Bcl-2 molecules, according to the expression degrees rated as low and high of the colorectal adenocarcinoma (*n* = 114).

With regard to the correlation of ECM genes IHC expression levels with the non-ECM molecules P53, Bcl-2, VEGF, KI-67, and EGFR, this study showed that FN-1 expression did not correlate with any ECM marker or non-ECM molecule studied. Expression of ITGA-3 showed a weak correlation with EGFR (*r* = 0.74; *P* = 0.000), and ITGB-5 expression displayed a regular correlation (*r* = 0.42; *P* = 0.000) with EGFR. MMP-2 expression did not correlate with the non-EMC molecules studied. MMP-9 was shown to have a strong expression correlation (*r* = 0.76; *P* = 0.000).  Table 11 shows the results of the distribution of the correlation Spearman coefficients between the ECM genes and the non-ECM molecules markers studied.

The results of RT-PCR and IHC methods in 114 patients with colorectal adenocarcinoma are shown in the Table 12, where differentially expressed extracellular matrix genes and the respective proteins are correlated. The findings of immunohistochemistry technique validated the correlation between transcript and protein. While RT-PCR is regarded as a tool which provides a genic screening the immunohistochemical technique allows the identification of the correspondent protein of the genes differentially expressed.

## 4. Discussion

### 4.1. The Possible Role of the ECM in CRC Dissemination

Carcinogenesis and tumor progression represent complex processes that involve a series of events traditionally characterized as a cascade of phenomena that require more investigation to be fully elucidated. It has been assumed that as a result of the cascade of carcinogenesis, progression, and dissemination, the initial transformation requires the tumor cell to be able to invade the surrounding tissues, which would characterize its malignant nature. Thus, it is necessary that these cells detach from their adhesive interactions in the epithelium, penetrate the basal membrane, degrade the ECM, and migrate to the subjacent interstitial stroma. At this point, the tumor cell enters the blood and lymphatic stream, acquiring systemic dissemination. In the intestine, particularly, the basal membrane separates the epithelial tissue from the conjunctive tissue, and a histopathological characteristic of intestinal tumors is the loss of the basal membrane integrity [24].

The ECM is composed of a large variety of structural molecules such as collagen, noncollagen glycoproteins, and proteoglycans that play a complex role in the regulation of cell behavior, influencing its development, growth, survival, migration, signal transduction, structure, and function [25, 26].

Thus, the degradation of elements constituting the basal membrane and ECM mediated by certain proteolytic enzymes, usually metalloproteinases, can represent a fundamental step in tumor progression and metastasis [4].

In recent decades, research in the field of cancer biology has focused extensively on the role of the ECM constituents during tumor progression. Some proteins located in specific domains of the ECM play a critical role in keeping the cells linked to matrix elements and the basal membrane, also participating in the matrix-cell signaling cascades. This information from the ECM is transmitted to the cells, mainly by means of integrin molecules, to activate, for example, cytokines, growth factors, and intracellular adaptor molecules. Thus, it can significantly affect many different processes such as cell cycle progression and cell migration and differentiation. The interaction between the biophysical properties of the cell and the ECM establishes a dynamic reciprocity, generating a sequence of reactions with a complex network of proteases, sulfatases, and possibly other enzymes to release and activate several signaling pathways in a very specific and localized manner. ECM homeostasis is, therefore, a delicate balance between the biosynthesis of proteins, its structural organization, biosignaling, and the degradation of its elements [7].

### 4.2. The Methods Used for Tracking and Identifying ECM Genes

The simplicity of PCR array allows it to be used for routine research, as it is a reliable tool to analyze the expression of a panel of genes specific to a particular pathology, offering high sensitivity and broad dynamic range. The use of the SuperArray Kit (PAHs-031A-24 Ambriex) for ECM and cell adhesion molecules allowed for the analysis of the expression of 84 genes important for cell-cell and cell-matrix interactions. This array contains ECM proteins including basal membrane, collagen, and gene constituents.

Using RT-PCR, it was possible to analyze in a quick, simple, and reliable manner the expression of a group of gene transcripts involved in the progression and dissemination of colorectal adenocarcinoma in several staging phases. Various studies have used this method in different types of malignant neoplastic diseases [18, 27] with regard to angiogenesis [19], apoptosis [20], and the cell cycle [28].

The TMA, a technique described by Kononen et al., in 1998, is widely accepted in the literature. This extremely simple concept consists of grouping a large number of tissue samples in a single paraffin block and allows for the study of the expression of molecular markers in a large scale with the use of stored material. TMAs have advantages over traditional cuts such as a reduction in reagents and time required to perform the reactions. The standardization of the reactions has facilitated comparative interpretation of research cases [29, 30].

The use of monoclonal antibodies with IHC examination in the TMA and in situ hybridization techniques allows for the detection of differential tissue protein expression corresponding to the gene (validation process of tracing techniques) in a simplified manner, as well as more elaborate technical standardization, hence minimizing the possibility of measurement biases.

### 4.3. The Results

In this study, an increase of the expression of integrins alpha 3 and beta 5 genes can be observed in advanced tumors locally or remotely in stages III and IV relative to stages I and II, which represent nonmetastatic tumors to lymph nodes and/or other sites, a fact confirmed by protein expression using the TMA technique. Other correlations such as histologic type and venous and neural invasion were also found to be significant, further reinforcing the possible role of integrins in tumor progression and dissemination in colorectal adenocarcinoma.

Integrin alpha 3 is usually expressed in normal tissues and in various tumors. Studies evaluating the expression of integrin alpha 3 in primary colon cancer and its respective metastases in the liver have shown that almost 27.5% of primary tumors presented with increased expression of integrin alpha 3 relative to the metastatic tumor. In the present study, while evaluating gene expression, there was a significant difference in the expression of integrin alpha 3, a result validated by the analysis of protein expression by the immunohistochemical method, and a greater expression was observed in the TNM III and IV groups than in the TNM I and II groups, thus suggesting a possible relation of integrin 3 with more advanced stages of colorectal cancer. Significant differences were also found in the ITGA-3 protein levels between the presence and absence of venous invasion; however, this fact was not supported by the gene expression levels using RT-PCR.

In 1999, Haler et al. studied the expression levels of integrins alpha 2, 3, 5, and 6 using IHC in lineages of liver metastatic colorectal carcinoma cells; an increased expression of integrins alpha 2 and alpha 3 was observed, particularly, with regard to the potential dissemination of CRC [31]. Jinka et al., in a recent study, observed the same results, including, however, a higher number of malignant tumors [7]. In the present study, the ITGB-5 gene showed significantly higher expression levels in the TNM III and IV stage groups compared with the TNM I and II groups; these data were confirmed by the results of the protein expression analysis using immunohistochemistry.

When comparing ITGB-5 gene expression with the degree of cell differentiation, a significant difference was observed in the grade III group compared with the grades I and II groups; however, when evaluating the protein expression of the ITGB-5 gene, there were no significant differences between the degrees of cell differentiation in the analyses.

A recent study, using cell cultures of human breast cancer and normal epithelial tissue, demonstrated a role of integrin beta 5 in tumor progression and invasion by changes in adhesion, the cell structure, and differentiation, when the inhibition of this integrin was found to significantly reduce breast carcinoma cell invasion [32]. It has also been reported that integrin expression levels may vary considerably between the normal and tumor tissue. In a certain way, integrins alpha v beta 5 and alpha v beta 6 are generally expressed in low levels or are nondetectable in normal human adult epithelium and are highly expressed in some tumors, correlating to more advanced stages of the disease [33].

It is possible that the increased expression of integrins alpha v beta 3 and alpha v beta 5 promotes the linking of tumor cells in the temporary matrix proteins such as vitronectin, fibrinogen, von Willebrand factor, osteopontin, and fibronectin that are deposited in the tumor microenvironment, facilitating the process of endothelial angiogenesis [34].

Among the studied genes, the overexpression of the following metalloproteinases in the tumor tissue can be correlated to at least one clinicopathological variable: MMP-1, MMP-2, MMP-9, MMP-11, and MMP-16.

MMP-2 and MMP-9 metalloproteinases, our research subjects, have been reported as essential in the tumor dissemination and progression process by many authors. These proteins degrade the main component of the basal membrane, which is type IV collagen. In several studies comparing MMP-2 expression levels in CRC and clinicopathological variables, a significance was observed regarding the strong expression of this enzyme in stages III and IV (TNM) [35, 36], tumor size and venous invasion, lymph node metastasis [35, 37], and distant metastasis [35, 36, 38].

MMP-2, called gelatinase A, in addition to type IV collagen, degrades other types of this protein such as V, VII, and X and also fibronectin, laminin, and elastin, which are components of the ECM [35]. Thus, MMP-2 expression has been investigated in several cancer types including colorectal adenocarcinoma, as MMP is significantly increased in the tumor tissue compared with the nontumor tissue [39].

In our study, there was a correlation of MMP-2 gene and protein expression levels with the clinicopathological variables such as mucinous histological type with signet ring cells and SOE adenocarcinoma; our results show that the MMP2 has potential as a prognostic CRC marker, in agreement with other published studies.

MMP9, known as gelatinase B, promotes the degradation of an important component of the basal membrane, type IV collagen, which is crucial for the invasion of malignant tumors from the proteolysis of ECM with CRC progression and metastasis [40]. Thus, there is substantial interest in the study of the MMP-9 expression in CRC as a prognostic marker.

Several studies in the literature have shown increased expression of MMP-9 in CRC with significance with regard to clinicopathological variables such as stages III and IV (TNM/Dukes C and D) [38–41], lymph node metastasis [40, 42], remote metastasis [37–40], peritumoral inflammatory infiltrate [43], and degree of cell differentiation II and III [38, 40, 41, 44, 45].

In this study, MMP-9 expression appeared significantly more frequently in the villous histological type that, according to the literature studies, shows a better prognosis than SOE adenocarcinomas [46].

### 4.4. Correlation of IHC Expression of the EMC Genes of Interest FN-1, ITGA-3, ITGB-5, MMP-2, and MMP-9 with the Non-ECM Molecules EGFR, VEGF, P53, Bcl-2, and KI-67

According to Viana et al., in 2013, ECM components interact with non-ECM molecules in CRC carcinogenesis, progression, and dissemination. One of the goals of our study was to evaluate the correlation of the expression of ECM components with that of P53, Bcl-2, KI-67, EGFR, and VEGF because it is known that proliferation, apoptosis, and cell migration are regulated by cell-cell interactions and ECM cell components. It is also worth noting that growth factors (e.g., EGF and VEGF) are usually stored in the ECM and can be activated and released after ECM modulation [12, 15]. In this study, we found that the correlation between MMP-9 and ITGA-3 genes with epithelial marker EGFR has been strong, whereas no relationship between the tumor expression of MMP-2, FN-1, and ITGB-5 with non-ECM molecules VEGF, KI-67, P53, and Bcl-2 could be demonstrated.

## 5. Conclusions

In CRCs, the overexpression of ITGA-3 and ITGB-5 genes and of their proteins was associated with lymph nodal dissemination stages and remote metastasis, whereas the overexpression of MMP-2 and MMP-9 genes and their proteins was associated with the mucinous and villous histological types, respectively. The epithelial marker EGFR (epidermal growth factor receptor) overactivity has been shown to be associated with the ECM genes MMP-9 and ITGA-3 expression.

## Figures and Tables

**Table 1:** Characteristics of the 114 patients included in the study.

Variables	*n*	%
Age		
<60 years >60 years	56 58	49.1 50.9
Gender		
Female Male	51 63	44.7 55.3
Primary tumor site		
Right colon Left colon Rectum	41 41 32	36.0 36.0 28.0
Synchronous tumor		
No Yes	112 2	98.2 1.8
Histological classification		
Adenocarcinoma SOE Adenocarcinoma mucinous Adenocarcinoma villous	81 18 15	71.0 15.8 13.2
Grading: cell differentiation		
Well differentiated Moderate Poor Undifferentiated	9 91 14 0	7.9 79.8 12.3 0
Venous invasion		
Absent Present	93 21	81.6 18.4
Lymphatic vessels invasion		
Absent Present	91 23	79.8 20.2
Perineural invasion		
Absent Present	106 8	93.0 7.0
Peritumoural lymphocyte infiltration		
Absent Present	21 93	18.4 81.6
Resection margin status		
Positive Negative	0 114	0 100.0
Lymph nodes dissected		
Median Range	17*	3–67
Tumor stage: TNM		
T1 T2 T3 T4	5 27 71 11	4.4 23.7 62.3 9.6
Nodal stage		
N0 N1 N2	67 25 22	58.8 21.9 19.3
Distant metastasis		
Absent Present	98 16	85.9 14.1
Site of distant metastasis		
Absent Liver Peritoneum Lungs Ovary	98 9 3 2 2	85.9 7.9 2.6 1.8 1.8
Clinical stage		
I II III IV	25 39 34 16	21.9 34.2 29.8 14.0

*28 patients had <12 lymph nodes dissected or analyzed.

**Table 2:** Distribution of expression levels of FN-1, ITGA-3, ITGB-5, MMP-2, and MMP-9 ECM genes, with significance levels of *P* < 0.05, fold change >2.0, and clinicopathological variables associated with genetic tracing by RT-PCR.

Gene	*P* value	Fold change	Clinicopathological parameter	Comparison
FN-1	0.022	−3.07	Age (years)	<60 × ≥60
ITGA-3	0.016	2.58	TNM	TNM III × TNM I
ITGB-5	0.04	−2.11	Degree of cell differentiation	GII × GI
	0.029	1.33	TNM	TNM III × TNM I
MMP-2	0.015	2.17	Histological type	Mucinous × tubular
	0.04	−1.2	Peritumoral lymphocyte infiltration	With × without
	0.039	−2.11	Age	>60 × ≤60
MMP-9	0.014	1.13	Histological type	Villous × tubular

**Table 3:** Distribution of the expression levels PER IHC of proteins corresponding to ITGB-5, ITGA-3, MMP-2, MMP-9, and FN-1 ECM genes and EGFR, VEGF, KI-67, P53, and Bcl-2 molecules as per the degree of cell differentiation of CRC (*n* = 114).

Grading tumor cell differentiation	EGFR		Total	*P* value
	High	Low		
I + II				0.159
*N*	63	37	100	
%	63.0%	37.0%	100.0%	
III				
*N*	6	8	14	
%	42.9%	57.1%	100.0%	
Total				
*N*	**69 **	**45 **	**114 **	
%	**60.5%**	**39.5%**	**100.0%**	

Grading tumor cell differentiation	VEGF		Total	*P *value
	High	Low		

I + II				0.778
*N*	49	51	100	
%	49.0%	51.0%	100.0%	
III				
*N*	6	8	14	
%	42.9%	57.1%	100.0%	
Total				
*N*	**55 **	**59 **	**114 **
%	**48.2%**	**51.8%**	**100.0%**

Grading tumor cell differentiation	KI-67		Total	*P *value
	High	Low		

I + II				0.093
*N*	46	54	100	
%	46.0%	54.0%	100.0%	
III				
*N*	3	11	14	
%	21.4%	78.6%	100.0%	
Total				
*N*	**49 **	**65 **	**114 **	
%	**43.0%**	**57.0%**	**100.0%**	

Grading tumor cell differentiation	P53		Total	*P *value
	High	Low		

I + II				0.778
*N*	41	59	100	
%	41.0%	59.0%	100.0%	
III				
*N*	5	9	14	
%	35.7%	64.3%	100.0%	
Total				
*N*	**46 **	**68**	**114**	
%	**40.4%**	**59.6%**	**100.0%**	

Grading tumor cell differentiation	Bcl-2		Total	*P *value
	High	Low		

I + II				1.000
*N*	50	50	100	
%	50.0%	50.0%	100.0%	
III				
*N*	7	7	14	
%	50.0%	50.0%	100.0%	

Grading tumor cell differentiation	ITGB-5		Total	*P *value
	High	Low		

I + II				0.394
*N*	57	43	100	
%	57.0%	43.0%	100.0%	
III				
*N*	6	8	14	
%	42.9%	57.1%	100.0%	
Total				
*N*	**63**	**51**	**114**	
%	**55.3%**	**44.7%**	**100.0%**	

Grading tumor cell differentiation	ITGA-3		Total	*P *value
	High	Low		

I + II				0.397
*N*	50	50	100	
%	50.0%	50.0%	100.0%	
III				
*N*	5	9	14	
%	35.7%	64.3%	100.0%	
Total				
*N*	**55**	**59**	**114**	
%	**48.2%**	**51.8%**	**100.0%**	

Grading tumor cell differentiation	MMP-2		Total	*P *value
	High	Low		

I + II				0.568
*N*	48	52	100	
%	48.0%	52.0%	100.0%	
III				
*N*	5	9	14	
%	35.7%	64.3%	100.0%	
Total				
*N*	**53**	**61**	**114**	
%	**46.5%**	**53.5%**	**100.0%**	

Grading tumor cell differentiation	MMP-9		Total	*P *value
	High	Low		

I + II				0.572
*N*	53	47	100	
%	53.0%	47.0%	100.0%	
III				
*N*	6	8	14	
%	42.9%	57.1%	100.0%	
Total				
*N*	**59**	**55**	**114**	
%	**51.8%**	**48.2%**	**100.0%**	

Grading tumor cell differentiation	FN-1		Total	*P *value
	High	Low		

I + II				0.225
*N*	73	27	100	
%	73.0%	27.0%	100.0%	
III				
*N*	8	6	14	
%	57.1%	42.9%	100.0%	
Total				
*N*	**81**	**33**	**114**	
%	**71.1%**	**28.9%**	**100.0%**	

**Table 4:** The distribution of expression levels per IHC of proteins corresponding to ITGB-5, ITGA-3, MMP-2, MMP-9, and FN-1 ECM genes and EGFR, VEGF, KI-67, P53, and Bcl-2 molecules as per the degree of TNM staging of CRC (*n* = 114).

TNM tumor stage	EGFR		Total	*P* value
	High	Low		
I + II				0.000
*N*	64	0	64	
%	100.0%	0.0%	100.0%	
III + IV				
*N*	5	45	50	
%	10.0%	90.0%	100.0%	
Total				
*N*	**69**	**45**	**114**	
%	**60.5%**	**39.5%**	**100.0%**	

TNM tumor stage	VEGF		Total	*P *value
	High	Low		

I + II				0.186
*N*	27	37	64	
%	42.2%	57.8%	100.0%	
III + IV				
*N*	28	22	50	
%	56.0%	44.0%	100.0%	
Total				
*N*	**55**	**59**	**114**
%	**48.2%**	**51.8%**	**100.0%**

TNM tumor stage	KI-67		Total	*P *value
	High	Low		

I + II				0.000
*N*	38	26	64	
%	59.4%	40.6%	100.0%	
III + IV				
*N*	11	39	50	
%	22.0%	78.0%	100.0%	
Total				
*N*	**49**	**65**	**114**	
%	**43.0%**	**57.0%**	**100.0%**	

Estadiamento	P53		Total	*P *value
	High	Low		

I + II				0.000
*N*	35	29	64	
%	54.7%	45.3%	100.0%	
III + IV				
*N*	11	39	50	
%	22.0%	78.0%	100.0%	
Total				
*N*	**46**	**68**	**114**	
%	**40.4%**	**59.6%**	**100.0%**	

TNM tumor stage	Bcl-2		Total	*P *value
	High	Low		

I + II				0.345
*N*	29	35	64	
%	45.3%	54.7%	100.0%	
III + IV				
*N*	28	22	50	
%	56.0%	44.0%	100.0%	
Total				
*N*	**57**	**57**	**114**	
%	**50.0%**	**50.0%**	**100.0%**	

TNM tumor stage	ITGB-5		Total	*P *value
	High	Low		

I + II				0.000
*N*	49	15	64	
%	76.6%	23.4%	100.0%	
III + IV				
*N*	14	36	50	
%	28.0%	72.0%	100.0%	
Total				
*N*	**63**	**51**	**114**	
%	**55.3%**	**44.7%**	**100.0%**	

TNM tumor stage	ITGA-3		Total	*P *value
	High	Low		

I + II				0.000
*N*	53	11	64	
%	82.8%	17.2%	100.0%	
III + IV				
*N*	2	48	50	
%	4.0%	96.0%	100.0%	
Total				
*N*	**55**	**59**	**114**	
%	**48.2%**	**51.8%**	**100.0%**	

TNM tumor stage	MMP-2		Total	*P *value
	High	Low		

I + II				0.572
*N*	28	36	64	
%	43.8%	56.3%	100.0%	
III + IV				
*N*	25	25	50	
%	50.0%	50.0%	100.0%	
Total				
*N*	**53**	**61**	**114**	
%	**46.5%**	**53.5%**	**100.0%**	

TNM tumor stage	MMP-9		Total	*P *value
	High	Low		

I + II				0.000
*N*	56	8	64	
%	87.5%	12.5%	100.0%	
III + IV				
*N*	3	47	50	
%	6.0%	94.0%	100.0%	
Total				
*N*	**59**	**55**	**114**	
%	**51.8%**	**48.2%**	**100.0%**	

TNM tumor stage	FN-1		Total	*P *value
	high	low		

I + II				0.677
*N*	44	20	64	
%	68.8%	31.3%	100.0%	
III + IV				
*N*	37	13	50	
%	74.0%	26.0%	100.0%	
Total				
*N*	**81**	**33**	**114**	
%	**71.1%**	**28.9%**	**100.0%**	

**Table 5:** The distribution of expression levels per IHC of proteins corresponding to ITGB-5, ITGA-3, MMP-2, MMP-9, and FN-1 ECM genes and EGFR, VEGF, KI-67, P53, and Bcl-2 molecules as per the peritumoral lymphocyte infiltrate in CRC (*n* = 114).

Peritumoral lymphocyte infiltrate	EGFR		Total	*P* value
	High	Low		
Absence				
*N*	13	8	21	1.000
%	61.9%	38.1%	100.0%	
Presence				
*N*	56	37	93	
%	60.2%	39.8%	100.0%	
Total				
*N*	**69**	**45**	**114**	
%	**60.5%**	**39.5%**	**100.0%**	

Peritumoral lymphocyte infiltrate	VEGF		Total	*P *value
	High	Low		

Absence				0.635
*N*	9	12	21	
%	42.9%	57.1%	100.0%	
Presence				
*N*	46	47	93	
%	49.5%	50.5%	100.0%	
Total				
*N*	**55**	**59**	**114**
%	**48.2%**	**51.8%**	**100.0%**

Peritumoral lymphocyte infiltrate	KI-67		Total	*P *value
	High	Low		

Absence				
*N*	11	10	21	0.343
%	52.4%	47.6%	100.0%	
Presence				
*N*	38	55	93	
%	40.9%	59.1%	100.0%	
Total				
*N*	**49**	**65**	**114**	
%	**43.0%**	**57.0%**	**100.0%**	

Peritumoral lymphocyte infiltrate	P53		Total	*P *value
	High	Low		

Absence				1.000
*N*	8	13	21	
%	38.1%	61.9%	100.0%	
Presence				
*N*	38	55	93	
%	40.9%	59.1%	100.0%	
Total				
*N*	**46**	**68**	**114**	
%	**40.4%**	**59.6%**	**100.0%**	

Peritumoral lymphocyte infiltrate	Bcl-2		Total	*P *value
	High	Low		

Absence				0.629
*N*	9	12	21	
%	42.9%	57.1%	100.0%	
Presence				
*N*	48	45	93	
%	51.6%	48.4%	100.0%	
Total				
*N*	**57**	**57**	**114**	
%	**50.0%**	**50.0%**	**100.0%**	

Peritumoral lymphocyte infiltrate	ITGB-5		Total	*P *value
	High	Low		

Absence				1.000
*N*	12	9	21	
%	57.1%	42.9%	100.0%	
Presence				
*N*	51	42	93	
%	54.8%	45.2%	100.0%	
Total				
*N*	**63**	**51**	**114**	
%	**55.3%**	**44.7%**	**100.0%**	

Peritumoral lymphocyte infiltrate	ITGA-3		Total	*P *value
	High	Low		

Absence				0.227
*N*	13	8	21	
%	61.9%	38.1%	100.0%	
Presence				
*N*	42	51	93	
%	45.2%	54.8%	100.0%	
Total				
*N*	**55**	**59**	**114**	
%	**48.2%**	**51.8%**	**100.0%**	

Peritumoral lymphocyte infiltrate	MMP-2		Total	*P *value
	High	Low		

Absence				1.000
*N*	10	11	21	
%	47.6%	52.4%	100.0%	
Presence				
*N*	43	50	93	
%	46.2%	53.8%	100.0%	
Total				
*N*	**53**	**61**	**114**	
%	**46.5%**	**53.5%**	**100.0%**	

Peritumoral lymphocyte infiltrate	MMP-9		Total	*P *value
	High	Low		

Absence				1.000
*N*	11	10	21	
%	52.4%	47.6%	100.0%	
Presence				
*N*	48	45	93	
%	51.6%	48.4%	100.0%	
Total				
*N*	**59**	**55**	**114**	
%	**51.8%**	**48.2%**	**100.0%**	

Peritumoral lymphocyte infiltrate	FN-1		Total	*P *value
	High	Low		

Absence				0.180
*N*	12	9	21	
%	57.1%	42.9%	100.0%	
Presence				
*N*	69	24	93	
%	74.2%	25.8%	100.0%	
Total				
*N*	**81**	**33**	**114**	
%	**71.1%**	**28.9%**	**100.0%**	

**Table 6:** The distribution of expression levels per IHC of proteins corresponding to ITGB-5, ITGA-3, MMP-2, and MMP9 ECM genes and FN-1 and EGFR, VEGF, KI-67, P53, and Bcl-2 molecules as per the presence of venous invasion in CRC (*n* = 114).

Venous invasion	EGFR		Total	*P* value
	High	Low		
Absence				0.000
*N*	65	28	93	
%	69.9%	30.1%	100.0%	
Presence				
*N*	4	17	21	
%	19.0%	81.0%	100.0%	
Total				
*N*	**69**	**45**	**114**	
%	**60.5%**	**39.5%**	**100.0%**	

Venous invasion	VEGF		Total	*P *value
	High	Low		

Absence				0.810
*N*	44	49	93	
%	47.3%	52.7%	100.0%	
Presence				
*N*	11	10	21	
%	52.4%	47.6%	100.0%	
Total				
*N*	**55**	**59**	**114**
%	**48.2%**	**51.8%**	**100.0%**

Venous invasion	KI-67		Total	*P *value
	High	Low		

Absence				0.055
*N*	44	49	93	
%	47.3%	52.7%	100.0%	
Presence				
*N*	5	16	21	
%	23.8%	76.2%	100.0%	
Total				
*N*	**49**	**65**	**114**	
%	**43.0%**	**57.0%**	**100.0%**	

Venous invasion	P53		Total	*P *value
	High	Low		

Absence				0.029
*N*	42	51	93	
%	45.2%	54.8%	100.0%	
Presence				
*N*	4	17	21	
%	19.0%	81.0%	100.0%	
Total				
*N*	**46**	**68**	**114**	
%	**40.4%**	**59.6%**	**100.0%**	

Venous invasion	Bcl-2		Total	*P *value
	High	Low		

Absence				1.000
*N*	46	47	93	
%	49.5%	50.5%	100.0%	
Presence				
*N*	11	10	21	
%	52.4%	47.6%	100.0%	
Total				
*N*	**57**	**57**	**114**	
%	**50.0%**	**50.0%**	**100.0%**	

Venous invasion	ITGB-5		Total	*P *value
	High	Low		

Absence				0.231
*N*	54	39	93	
%	58.1%	41.9%	100.0%	
Presence				
*N*	9	12	21	
%	42.9%	57.1%	100.0%	
Total				
*N*	**63**	**51**	**114**	
%	**55.3%**	**44.7%**	**100.0%**	

Venous invasion	ITGA-3		Total	*P *value
	High	Low		

Absence				0.000
*N*	52	41	93	
%	55.9%	44.1%	100.0%	
Presence				
*N*	3	18	21	
%	14.3%	85.7%	100.0%	
Total				
*N*	**55**	**59**	**114**	
%	**48.2%**	**51.8%**	**100.0%**	

Venous invasion	MMP-2		Total	*P *value
	High	Low		

Absence				0.631
*N*	42	51	93	
%	45.2%	54.8%	100.0%	
Presence				
*N*	11	10	21	
%	52.4%	47.6%	100.0%	
Total				
*N*	**53**	**61**	**114**	
%	**46.5%**	**53.5%**	**100.0%**	

Venous invasion	MMP-9		Total	*P *value
	High	Low		

Absence				0.001
*N*	55	38	93	
%	59.1%	40.9%	100.0%	
Presence				
*N*	4	17	21	
%	19.0%	81.0%	100.0%	
Total				
*N*	**59**	**55**	**114**	
%	**51.8%**	**48.2%**	**100.0%**	

Venous invasion	FN-1		Total	*P *value
	High	Low		

Absence				0.006
*N*	61	32	93	
%	65.6%	34.4%	100.0%	
Presence				
*N*	20	1	21	
%	95.2%	4.8%	100.0%	
Total				
*N*	**81**	**33**	**114**	
%	**71.1%**	**28.9%**	**100.0%**	

**Table 7:** The distribution of expression levels per IHC of proteins corresponding to ITGB-5, ITGA-3, MMP-2, MMP-9, and FN-1 ECM genes and EGFR, VEGF, Ki-67, P53, and Bcl-2 molecules as per the presence of perineural invasion in CRC (*n* = 114).

Perineural invasion	EGFR		Total	*P* value
	High	Low		
Absence				0.260
*N*	66	40	106	
%	62.3%	37.7%	100.0%	
Presence				
*N*	3	5	8	
%	37.5%	62.5%	100.0%	
Total				
*N*	**69**	**45**	**114**	
%	**60.5%**	**39.5%**	**100.0%**	

Perineural invasion	VEGF		Total	*P *value
	High	Low		

Absence				0.027
*N*	48	58	106	
%	45.3%	54.7%	100.0%	
Presence				
*N*	7	1	8	
%	87.5%	12.5%	100.0%	
Total				
*N*	**55**	**59**	**114**
%	**48.2%**	**51.8%**	**100.0%**

Perineural invasion	KI-67		Total	*P *value
	High	Low		

Absence				0.462
*N*	47	59	106	
%	44.3%	55.7%	100.0%	
Presence				
*N*	2	6	8	
%	25.0%	75.0%	100.0%	
Total				
*N*	**49**	**65**	**114**	
%	**43.0%**	**57.0%**	**100.0%**	

Perineural invasion	P53		Total	*P *value
	High	Low		

Absence				0.470
*N*	44	62	106	
%	41.5%	58.5%	100.0%	
Presence				
*N*	2	6	8	
%	25.0%	75.0%	100.0%	
Total				
*N*	**46**	**68**	**114**	
%	**40.4%**	**59.6%**	**100.0%**	

Perineural invasion	Bcl-2		Total	*P *value
	High	Low		

Absence				0.716
*N*	52	54	106	
%	49.1%	50.9%	100.0%	
Presence				
*N*	5	3	8	
%	62.5%	37.5%	100.0%	
Total				
*N*	**57**	**57**	**114**	
%	**50.0%**	**50.0%**	**100.0%**	

Perineural invasion	ITGB-5		Total	*P *value
	High	Low		

Absence				0.463
*N*	60	46	106	
%	56.6%	43.4%	100.0%	
Presence				
*N*	3	5	8	
%	37.5%	62.5%	100.0%	
Total				
*N*	**63**	**51**	**114**	
%	**55.3%**	**44.7%**	**100.0%**	

Perineural invasion	ITGA-3		Total	*P *value
	High	Low		

Absence				0.717
*N*	52	54	106	
%	49.1%	50.9%	100.0%	
Presence				
*N*	3	5	8	
%	37.5%	62.5%	100.0%	
Total				
*N*	**55**	**59**	**114**	
%	**48.2%**	**51.8%**	**100.0%**	

Perineural invasion	MMP-2		Total	*P *value
	High	Low		

Absence				0.999
*N*	49	57	106	
%	46.2%	53.8%	100.0%	
Presence				
*N*	4	4	8	
%	50.0%	50.0%	100.0%	
Total				
*N*	**53**	**61**	**114**	
%	**46.5%**	**53.5%**	**100.0%**	

Perineural invasion	MMP-9		Total	*P *value
	High	Low		

Absence				0.479
*N*	56	50	106	
%	52.8%	47.2%	100.0%	
Presence				
*N*	3	5	8	
%	37.5%	62.5%	100.0%	
Total				
*N*	**59**	**55**	**114**	
%	**51.8%**	**48.2%**	**100.0%**	

Perineural invasion	FN-1		Total	*P *value
	High	Low		

Absence				0.102
*N*	73	33	106	
%	68.9%	31.1%	100.0%	
Presence				
*N*	8	0	8	
%	100.0%	0.0%	100.0%	
Total				
*N*	**81**	**33**	**114**	
%	**71.1%**	**28.9%**	**100.0%**	

**Table 8:** The distribution of expression levels per IHC of proteins corresponding to ITGB-5, ITGA-3, MMP-2, MMP-9, and FN-1 ECM genes and EGFR, VEGF, KI-67, P53, and Bcl-2 molecules as per the villous and tubular types of CRC (*n* = 114).

Tumor histologic type: tubular × villous	EGFR		Total	*P* value
	High	Low		
Tubular				0.579
*N*	52	29	81	
%	64.2%	35.8%	100.0%	
Villous				
*N*	9	7	16	
%	56.3%	43.8%	100.0%	
Total				
*N*	**61**	**36**	**97**	
%	**62.9%**	**37.1%**	**100.0%**	

Tumor histologic type: tubular × villous	VEGF		Total	*P *value
	High	Low		

Tubular				1.000
*N*	41	40	81	
%	50.6%	49.4%	100.0%	
Villous				
*N*	8	8	16	
%	50.0%	50.0%	100.0%	
Total				
*N*	**49**	**48**	**97**
%	**50.5%**	**49.5%**	**100.0%**

Tumor histologic type: tubular × villous	KI-67		Total	*P *value
	High	Low		

Tubular				0.783
*N*	36	45	81	
%	44.4%	55.6%	100.0%	
Villous				
*N*	6	10	16	
%	37.5%	62.5%	100.0%	
Total				
*N*	**42**	**55**	**97**	
%	**43.3%**	**56.7%**	**100.0%**	

Tumor histologic type: tubular × villous	P53		Total	*P *value
	High	Low		

Tubular				1.000
*N*	32	49	81	
%	39.5%	60.5%	100.0%	
Villous				
*N*	6	10	16	
%	37.5%	62.5%	100.0%	
Total				
*N*	**38**	**59**	**97**	
%	**39.2%**	**60.8%**	**100.0%**	

Tumor histologic type: tubular × villous	Bcl-2		Total	*P *value
	High	Low		

Tubular				0.277
*N*	37	44	81	
%	45.7%	54.3%	100.0%	
Villous				
*N*	10	6	16	
%	62.5%	37.5%	100.0%	
Total				
*N*	**47**	**50**	**97**	
%	**48.5%**	**51.5%**	**100.0%**	

Tumor histologic type: tubular × villous	ITGB-5		Total	*P *value
	High	Low		

Tubular				1.000
*N*	44	37	81	
%	54.3%	45.7%	100.0%	
Villous				
*N*	9	7	16	
%	56.3%	43.8%	100.0%	
Total				
*N*	**53**	**44**	**97**	
%	**54.6%**	**45.4%**	**100.0%**	

Tumor histologic type: tubular × villous	ITGA-3		Total	*P *value
	High	Low		

Tubular				1.000
*N*	40	41	81	
%	49.4%	50.6%	100.0%	
Villous				
*N*	8	8	16	
%	50.0%	50.0%	100.0%	
Total				
*N*	**48**	**49**	**97**	
%	**49.5%**	**50.5%**	**100.0%**	

Tumor histologic type: tubular × villous	MMP-2		Total	*P *value
	High	Low		

Tubular				0.585
*N*	44	37	81	
%	54.3%	45.7%	100.0%	
Villous				
*N*	7	9	16	
%	43.8%	56.3%	100.0%	
Total				
*N*	**51**	**46**	**97**	
%	**52.6%**	**47.4%**	**100.0%**	

Tumor histologic type: tubular × villous	MMP-9		Total	*P *value
	High	Low		

Tubular				0.000
*N*	49	32	81	
%	60.5%	39.5%	100.0%	
Villous				
*N*	1	15	16	
%	6.3%	93.8%	100.0%	
Total				
*N*	**50**	**47**	**97**	
%	**51.5%**	**48.5%**	**100.0%**	

Tumor histologic type: tubular × villous	FN-1		Total	*P *value
	High	Low		

Tubular				1.000
*N*	57	24	81	
%	70.4%	29.6%	100.0%	
Villous				
*N*	11	5	16	
%	68.8%	31.3%	100.0%	
Total				
*N*	**68**	**29**	**97**	
%	**70.1%**	**29.9%**	**100.0%**	

**Table 9:** The distribution of expression levels per IHC of proteins corresponding to ITGB-5, ITGA-3, MMP-2, MMP-9, and FN-1 ECM genes and EGFR, VEGF, KI-67, P53, and Bcl-2 molecules as per mucinous and tubular tumor types of CRC (*n* = 114).

Tumor histologic type: mucinous × tubular	EGFR		Total	*P* value
	High	Low		
Mucinous				0.273
*N*	8	9	17	
%	47.1%	52.9%	100.0%	
Tubular				
*N*	52	29	81	
%	64.2%	35.8%	100.0%	
Total				
*N*	**60**	**38**	**98**	
%	**61.2%**	**38.8%**	**100.0%**	

Tumor histologic type: mucinous × tubular	VEGF		Total	*P *value
	High	Low		

Mucinous				0.294
*N*	6	11	17	
%	35.3%	64.7%	100.0%	
Tubular				
*N*	41	40	81	
%	50.6%	49.4%	100.0%	
Total				
*N*	**47**	**51**	**98**
%	**48.0%**	**52.0%**	**100.0%**

Tumor histologic type: mucinous × tubular	KI-67		Total	*P *value
	High	Low		

Mucinous				1.000
*N*	7	10	17	
%	41.2%	58.8%	100.0%	
Tubular				
*N*	36	45	81	
%	44.4%	55.6%	100.0%	
Total				
*N*	**43**	**55**	**98**	
%	**43.9%**	**56.1%**	**100.0%**	

Tumor histologic type: mucinous × tubular	P53		Total	*P *value
	High	Low		

Mucinous				0.596
*N*	8	9	17	
%	47.1%	52.9%	100.0%	
Tubular				
*N*	32	49	81	
%	39.5%	60.5%	100.0%	
Total				
*N*	**40**	**58**	**98**	
%	**40.8%**	**59.2%**	**100.0%**	

Tumor histologic type: mucinous × tubular	Bcl-2		Total	*P *value
	High	Low		

Mucinous				0.425
*N*	10	7	17	
%	58.8%	41.2%	100.0%	
Tubular				
*N*	37	44	81	
%	45.7%	54.3%	100.0%	
Total				
*N*	**47**	**51**	**98**	
%	**48.0%**	**52.0%**	**100.0%**	

Tumor histologic type: mucinous × tubular	ITGB-5		Total	*P *value
	High	Low		

Mucinous				0.793
*N*	10	7	17	
%	58.8%	41.2%	100.0%	
Tubular				
*N*	44	37	81	
%	54.3%	45.7%	100.0%	
Total				
*N*	**54**	**44**	**98**	
%	**55.1%**	**44.9%**	**100.0%**	

Tumor histologic type: mucinous × tubular	ITGA-3		Total	*P *value
	High	Low		

Mucinous				0.600
*N*	7	10	17	
%	41.2%	58.8%	100.0%	
Tubular				
*N*	40	41	81	
%	49.4%	50.6%	100.0%	
Total				
*N*	**47**	**51**	**98**	
%	**48.0%**	**52.0%**	**100.0%**	

Tumor histologic type: mucinous × tubular	MMP-2		Total	*P *value
	High	Low		

Mucinous				0.001
*N*	2	15	17	
%	11.8%	88.2%	100.0%	
Tubular				
*N*	44	37	81	
%	54.3%	45.7%	100.0%	
Total				
*N*	**46**	**52**	**98**	
%	**46.9%**	**53.1%**	**100.0%**	

Tumor histologic type: mucinous × tubular	MMP-9		Total	*P *value
	High	Low		

Mucinous				0.596
*N*	9	8	17	
%	52.9%	47.1%	100.0%	
Tubular				
*N*	49	32	81	
%	60.5%	39.5%	100.0%	
Total				
*N*	**58**	**40**	**98**	
%	**59.2%**	**40.8%**	**100.0%**	

Tumor histologic type: mucinous × tubular	FN-1		Total	*P *value
	High	Low		

Mucinous				0.771
*N*	13	4	17	
%	76.5%	23.5%	100.0%	
Tubular				
*N*	57	24	81	
%	70.4%	29.6%	100.0%	
Total				
*N*	**70**	**28**	**98**	
%	**71.4%**	**28.6%**	**100.0%**	

**Table 10:** The distribution of expression levels per IHC of proteins corresponding to ITGB-5, ITGA-3, MMP-2, MMP9, and FN-1 ECM genes and EGFR, VEGF, KI-67, P53, and Bcl-2 molecules as per the degrees of high and low expression of CRC (*n* = 114).

Markers	Low expression	High expression
	*n* (%)	*n* (%)
FN-1	81 (71.1)	33 (28.9)
ITGA-3	55 (48.2)	59 (51.8)
ITGB-5	63 (55.3)	51 (44.7)
MMP-2	53 (46.5)	61 (53.5)
MMP-9	59 (51.8)	55 (48.2)
P53	46 (40.4)	68 (59.6)
Bcl-2	57 (50.0)	57 (50.0)
KI-67	49 (43.0)	65 (57.0)
EGFR	69 (60.5)	45 (39.5)
VEGF	55 (48.2)	59 (51.8)

**Table 11:** The distribution of Spearman (*r*) correlation coefficients, two-tailed model for significant associations (*P* < 0.05) between the immunohistochemical expressions of ECM genes FN-1, ITGA-3, ITGB-5, MMP-2, and MMP-9 and epithelial markers EGFR, VEGF, KI-67, P53, and Bcl-2 in CRC (*n* = 114).

	FN-1	ITGA-3	ITGB-5	MMP-2	MMP-9	EGFR	VEGF	KI-67	P53	Bcl-2
FN1	1	—	—	—	—	—	—	—	—	—
ITGA-3	—	1	0.48	—	0.65	**0.74**	—	—	—	—
ITGB-5	—	0.48	1	—	0.43	0.42	—	—	—	—
MMP-2	—	—	—	1			—	−0.20	−0.01	—
MMP-9	—	0.65	0.43	—	1	**0.76**	—	0.30	0.22	—
EGFR	—	**0.74**	0.42	—	**0.76**	1	—	0.37	0.33	—
VEGF	—	—	—	—			1	—	—	0.33
KI-67	—	—	—	—	0.30	0.37	—	1	0.87	—
P53	—	—	—	−0.19	0.22	0.33	—	0.87	1	—
Bcl-2	—	—	—	—			0.33		—	1

0 = no correlation; 0–0.25 = weak; 0.25–0.50 = regular; 0.50–0.75 = moderate; >0.75 = strong; 1 = perfect correlation.

**Table 12:** Real-time PCR and immunohistochemistry correlation of differentially expressed extracellular matrix genes in 114 patients with colorectal adenocarcinoma, *P* < 0.05.

Genes	Classification	RT-PCR	RT-PCR	Parameters analysed	IHC	IHC
		Fold change	*P* value		Validation	*P* value
MMP-2	ECM proteases	2.17	0.01	Mucinous × tubular	No	0.001
		−1.2	0.04	Peritumoral lymphocyte infiltrate +/−	No	1.000
		−2.11	0.03	Age (>60 yr × <60 yr)	No	0.000

FN-1	Other adhesion molecules/collagens and ECM structural constituents	−3.07	0.02	AGE	No	1.000

ITGB-5	Transmembrane molecules/cell-matrix adhesion	2.11	0.04	Grade cell differentiation: (I, II × III, IV)	No	0.394
		1.33	0.02	TNM (I, II × III, IV)	Yes	0.000

ITGA-3	Transmembrane molecules	2.58	0.01	TNM (I, II × III, IV)	Yes	0.000

MMP-9	ECM proteases	1.13	0.01	Villous × tubular	Yes	0.000

IHC: immunohistochemistry; RT-PCR: reverse transcription polymerase.
